# Supplementary material for: College openings in the United States increase mobility and COVID-19 incidence
Source: PLoS One. 2022 Aug 29;17(8):e0272820. doi: 10.1371/journal.pone.0272820 (PMC9423614; doi:10.1371/journal.pone.0272820)
Supplement: S1 Fig — Counties were included in the CDC sample if the ratio of cumulative cases during our study period in the CDC line files relative to USAFacts was greater than 0.95 and the correlation in the 30-day cumulative case count time series was greater than 0.95. (PDF) [file pone.0272820.s001.pdf]

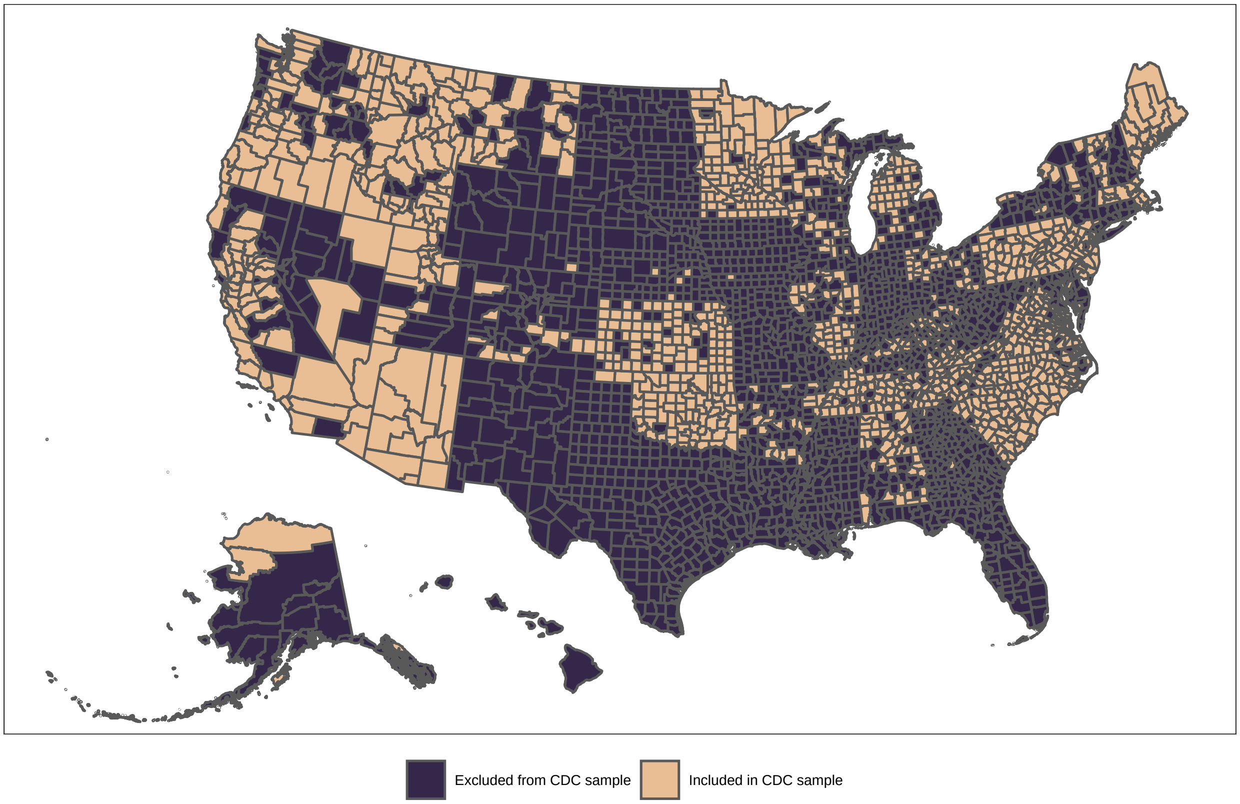

Figure 1: **Geographic distribution of counties that are included in the CDC sample.** Counties were included in the CDC sample if the ratio of cumulative cases during our study period in the CDC line files relative to USAFacts was greater than 0.95 and the correlation in the 30-day cumulative case count time series was greater than 0.95. Source: TIGRIS and authors' analysis.
