# Supplementary material for: College openings in the United States increase mobility and COVID-19 incidence
Source: PLoS One. 2022 Aug 29;17(8):e0272820. doi: 10.1371/journal.pone.0272820 (PMC9423614; doi:10.1371/journal.pone.0272820)
Supplement: S2 Fig — Colleges and universities are more prevalent in the eastern half of the United States, while colleges in the western half were more likely to reopen for online teaching. (PDF) [file pone.0272820.s002.pdf]

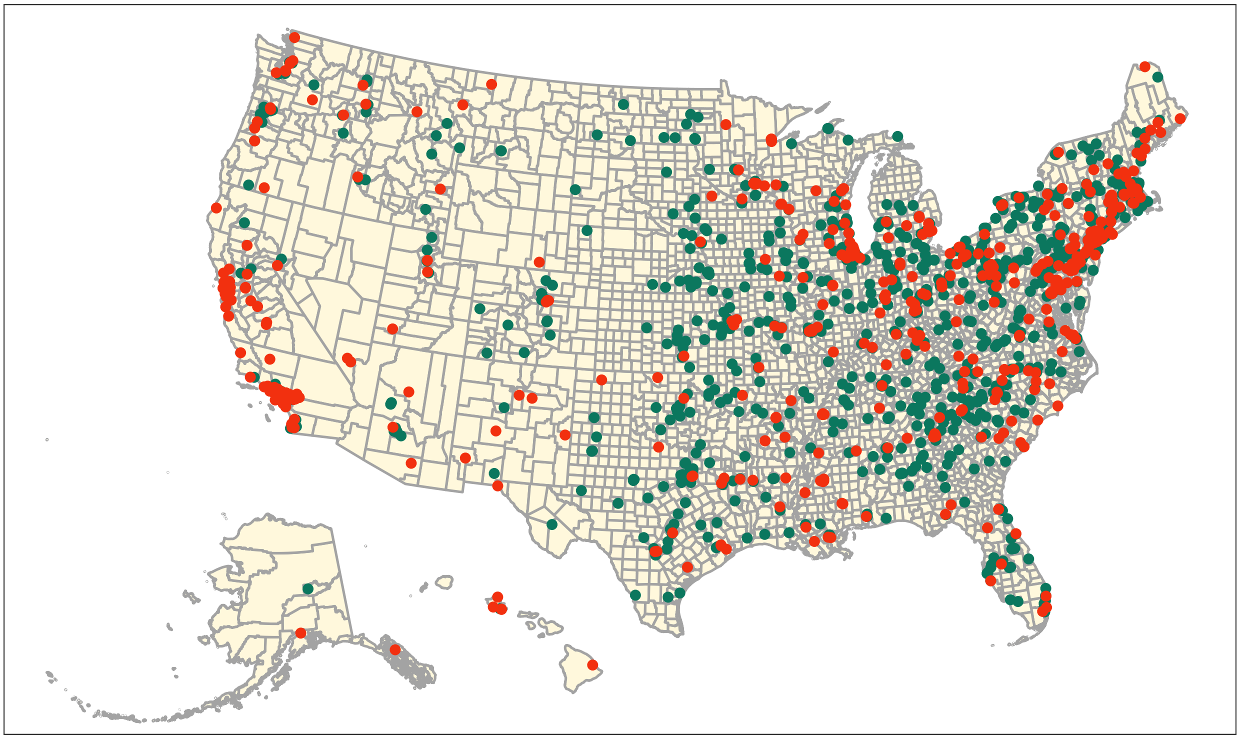

Teaching modality ● Primarily in−person ● Primarily online

Figure 2: **Geographic distribution of colleges and universities in sample by teaching modality.** Colleges and universities are more prevalent in the eastern half of the United States, while colleges in the western half were more likely to reopen for online teaching. Source: TIGRIS and authors' analysis.
