# Supplementary material for: College openings in the United States increase mobility and COVID-19 incidence
Source: PLoS One. 2022 Aug 29;17(8):e0272820. doi: 10.1371/journal.pone.0272820 (PMC9423614; doi:10.1371/journal.pone.0272820)
Supplement: S3 Fig — COVID-19 incidence rose for the 10-19 and 20-29 year old age groups following the resumption of classes, although the increase faded over time for both age groups (a), while cases requiring hospitalization (b) and ICU care (c) followed less precisely estimated patterns with a decreasing time trend throughout the study period. Mortality due to COVID-19 was stable throughout the study period as well for all age groups (d). (PDF) [file pone.0272820.s003.pdf]

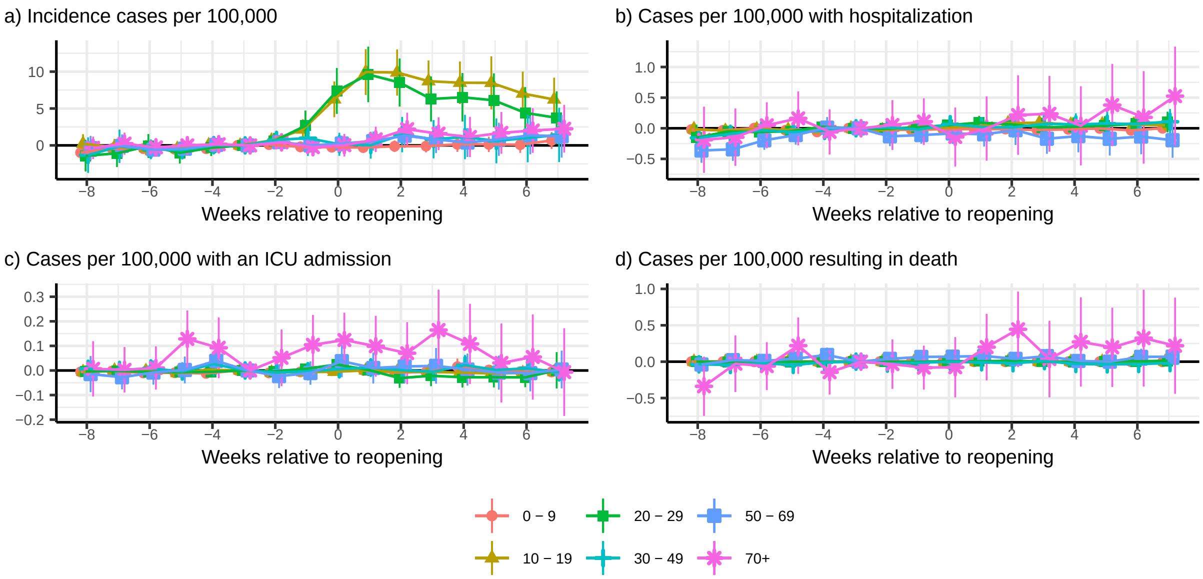

Figure 3: **Age-specific event studies**. COVID-19 incidence rose for the 10-19 and 20-29 year old age groups following the resumption of classes, although the increase faded over time for both age groups (a), while cases requiring hospitalization (b) and ICU care (c) followed less precisely estimated patterns with a decreasing time trend throughout the study period. Mortality due to COVID-19 was stable throughout the study period as well for all age groups (d).
