# Supplementary material for: College openings in the United States increase mobility and COVID-19 incidence
Source: PLoS One. 2022 Aug 29;17(8):e0272820. doi: 10.1371/journal.pone.0272820 (PMC9423614; doi:10.1371/journal.pone.0272820)
Supplement: S4 Fig — On-campus mobility increased for all teaching modalities (a), while COVID-19 incidence rose for “Fully in-person” (χ2(8) = 30.16, p < 0.001), “Primarily in-person” (χ2(8) = 27.79, p < 0.001), and “Primarily online” (χ2(8) = 21.25, p = 0.007) teaching modalities (b). CDC data (c) indicated that there were increases in COVID-19 incidence for “Hybrid” reopenings (χ2(8) = 26.52, p < 0.001). Hospitalization rates rose following “Fully in-person” (χ2(8) = 16.03, p = 0.042) reopenings (d). ICU admissions increased following reopening for “Hybrid” (χ2(8) = 21.43, p = 0.006) reopenings (e). Deaths did not increase for any reopening modality (f). Rt was significantly different following reopening for “Primarily in-person” (χ2(8) = 23.52, p = 0.003), “Hybrid” (χ2(8) = 25.66, p = 0.001), and “Primarily online” (χ2(8) = 25.82, p = 0.001) reopenings (g). (PDF) [file pone.0272820.s004.pdf]

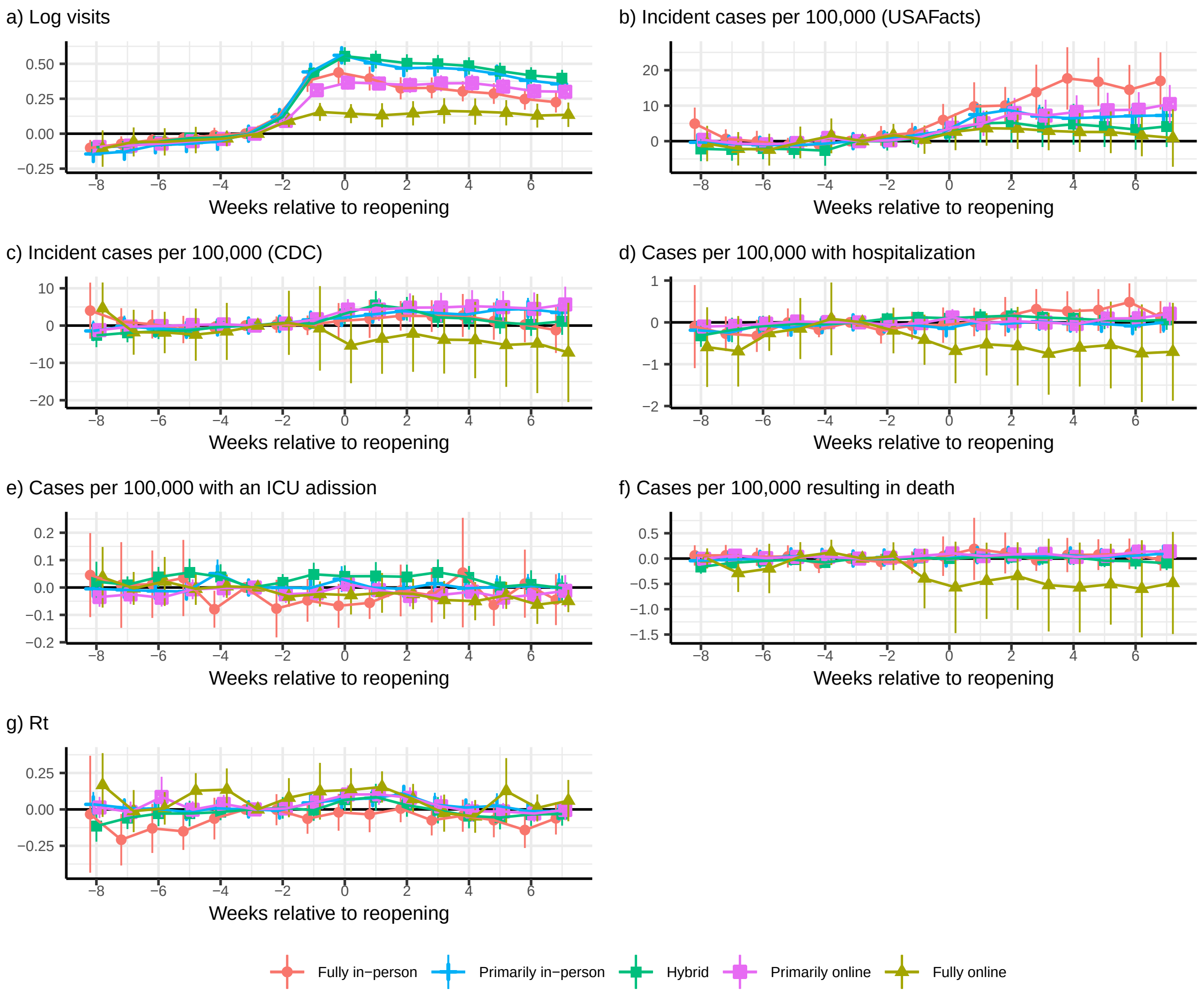

Figure 4: **Teaching modality-specific event studies.** On-campus mobility increased for all teaching modalities (a), while COVID-19 incidence rose for "Fully in-person" ($\chi^2(8) = 30.16$, $p < 0.001$), "Primarily in-person" ($\chi^2(8) = 27.79$, $p < 0.001$), and "Primarily online" ($\chi^2(8) = 21.25$, $p = 0.007$) teaching modalities (b). CDC data (c) indicated that there were increases in COVID-19 incidence for "Hybrid" reopenings ($\chi^2(8) = 26.52$, $p < 0.001$). Hospitalization rates rose following "Fully in-person" ($\chi^2(8) = 16.03$, $p = 0.042$) reopenings (d). ICU admissions increased following reopening for "Hybrid" ($\chi^2(8) = 21.43$, $p = 0.006$) reopenings (e). Deaths did not increase for any reopening modality (f). R_t was significantly different following reopening for "Primarily in-person" ($\chi^2(8) = 23.52$, $p = 0.003$), "Hybrid" ($\chi^2(8) = 25.66$, $p = 0.001$), and "Primarily online" ($\chi^2(8) = 25.82$, $p = 0.001$) reopenings (g).
