# Supplementary material for: College openings in the United States increase mobility and COVID-19 incidence
Source: PLoS One. 2022 Aug 29;17(8):e0272820. doi: 10.1371/journal.pone.0272820 (PMC9423614; doi:10.1371/journal.pone.0272820)
Supplement: S5 Fig — Counties were assigned on the basis of the earliest and, if necessary, largest college or university in each county. (PDF) [file pone.0272820.s005.pdf]

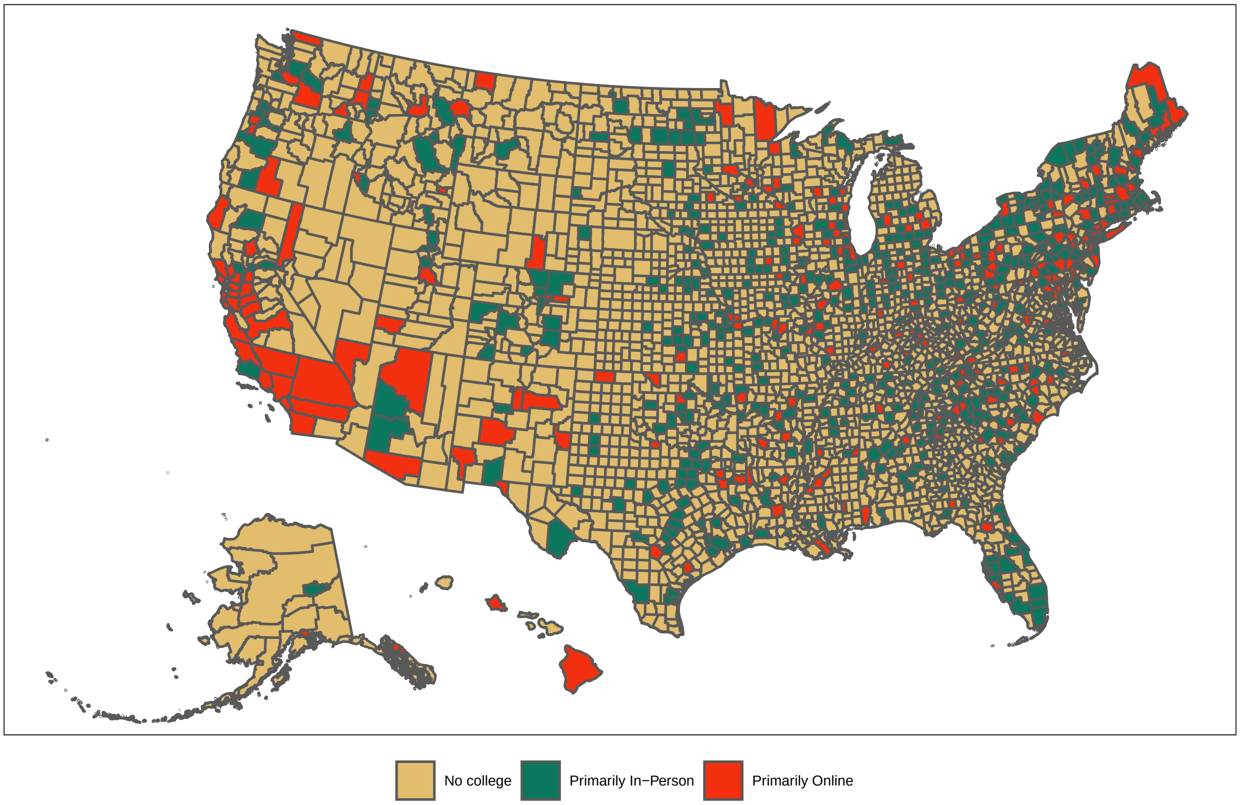

Figure 5: **Geographic distribution of teaching assignments.** Counties were assigned on the basis of the earliest and, if necessary, largest college or university in each county. Source: TIGRIS and authors' analysis.
