# Supplementary material for: College openings in the United States increase mobility and COVID-19 incidence
Source: PLoS One. 2022 Aug 29;17(8):e0272820. doi: 10.1371/journal.pone.0272820 (PMC9423614; doi:10.1371/journal.pone.0272820)
Supplement: S1 Appendix — (PDF) [file pone.0272820.s006.pdf]

College Openings, Mobility, and the Incidence of COVID-19

Martin S. Andersen, Ana I. Bento, Anirban Basu, Christopher R. Marsicano, Kosali Simon

July 28, 2022

1 Additional Methods

1.1 Effective reproductive number ($R(t)$)

We used the daily cases, incubation period, and serial interval previously estimated[1]. This allowed us to estimate the effective reproduction number for each county. The effective reproduction number $R(t)$ represents the mean number of secondary cases generated by a primary infector at time t[2–5]. This measure is useful to track the effectiveness of performed control measures, which aims to push it below the epidemic threshold (corresponding to $R(t) = 1$). $R(t)$ incorporates factors affecting the spread of the epidemic (e.g., individual's behavior and susceptible depletion).

To estimate $R(t)$, we use the same methodology described previously[4–6] to distinguish between locally acquired and imported cases. Thus, we assume that the daily number of new cases (date of symptom onset) with locally acquired infection $L(t)$ can be approximated by a Poisson distribution

$$C(t)Pois(R_t \sum_{s=1}^{t} \phi(s)C(t-s)) \quad (1)$$

where $C(t)$ is the number of new cases (either locally acquired or imported) at time t (date of symptom onset), $R(t)$ is the effective reproduction number at time t and is the generation time distribution. To estimate the time between consecutive generations of cases, we adopted the serial interval (which measures the time difference between the symptom onset of the infectors and of their infected) estimated from the literature[1], namely a gamma distribution with mean 5.0 days and standard deviation 3.4 days (shape=4.87, rate=0.65).

The likelihood λ of the observed time series cases from day 1 to T can be written as:

$$\lambda = \Pi_{t=1}^{T} P\left(C(t)Pois(R_t \sum_{s=1}^{t} \phi(s)C(t-s))\right) \quad (2)$$

where $P(x, y)$ is the Poisson density distribution of observing x events, given the parameter y.

We then use Metropolis-Hastings MCMC sampling to estimate the posterior distribution of $R(t)$. The Markov chains were run for 100,000 iterations, considering a burn-in period of 10,000 steps, and assuming non-informative prior distributions of $R(t)$ (flat distribution in the range (0-1000]). Convergence was checked by visual inspection by running multiple chains starting from different starting points.

1.2 Constructing exposure measures

We constructed a measure of a colleges exposure to different geographic areas using movement data from 2020. For each county and college campus we estimated the average number of devices from the source county on each campus by week and computed the change from 20 to 14 days before reopening to 0 to 6 days after reopening. Because in some cases the net flow went in the opposite direction, we truncated the change in devices at 0. We use this change in mobility as our estimate of the flow of devices from a source county to a college campus.

We then estimated, for each county, the 7-day incidence of COVID-19 per 100,000 people ending 14 days before campus reopened. Finally, we used the truncated number of devices moving from each county to a given campus to construct the weighted average of all counties that had net movement towards a college campus. The resulting exposure metric is the average 7-day incidence per 100,000 from source counties to a college campus for the period ending two weeks before campus reopened.

References

[1] Du Z, Xu X, Wu Y, Wang L, Cowling BJ, Meyers LA. Serial interval of COVID-19 among publicly reported confirmed cases. Emerging infectious diseases. 2020;26(6):1341.

[2] Keeling MJ, Rohani P. Modeling Infectious Diseases in Humans and Animals. Princeton University Press; 2011.

[3] Van Kerkhove MD, Bento AI, Mills HL, Ferguson NM, Donnelly CA. A review of epidemiological parameters from Ebola outbreaks to inform early public health decision-making. Scientific Data. 2015;2(1):150019. doi:10.1038/sdata.2015.19.

[4] Liu QH, Ajelli M, Aleta A, Merler S, Moreno Y, Vespignani A. Measurability of the Epidemic Reproduction Number in Data-Driven Contact Networks. Proceedings of the National Academy of Sciences. 2018;115(50):12680–12685. doi:10/ggfn24.

[5] Liu QH, Bento AI, Yang K, Zhang H, Yang X, Merler S, et al. The COVID-19 outbreak in Sichuan, China: Epidemiology and impact of interventions. PLOS Computational Biology. 2021;16(12):1–14. doi:10.1371/journal.pcbi.1008467.

[6] Cori A, Ferguson NM, Fraser C, Cauchemez S. A New Framework and Software to Estimate Time-Varying Reproduction Numbers During Epidemics. American Journal of Epidemiology. 2013;178(9):1505–1512. doi:10/f5gwtn.
