# Supplementary material for: College openings in the United States increase mobility and COVID-19 incidence
Source: PLoS One. 2022 Aug 29;17(8):e0272820. doi: 10.1371/journal.pone.0272820 (PMC9423614; doi:10.1371/journal.pone.0272820)
Supplement: S2 Appendix — (PDF) [file pone.0272820.s007.pdf]

College Openings, Mobility, and the Incidence of COVID-19

Martin S. Andersen, Ana I. Bento, Anirban Basu, Christopher R. Marsicano, Kosali Simon

July 28, 2022

2 Robustness checks and alternative specifications

We discuss our robustness checks, which are presented in table 7 below. The first row of table 7 repeats our baseline results as a reference.

2.1 Restricting to counties with a college

Restricting our sample to counties with a college or university reduces the magnitude of our estimates and leaves only the increase in mobility and the increase in COVID-19 incidence resulting in an ICU admission as statistically significant. However, except for incidence using the CDC data we are also unable to rule out our baseline point estimates using the restricted sample.

2.2 Restricting to counties with one or no college

Restricting our sample to counties with only single college or university or no college or university demonstrates the robustness of our results to these counties for which we can more precisely define treatment. Our results for disease incidence and R_t are all statistically significant and all of our estimates are comparable in magnitude to our baseline results. These results are suggestive that there is no significant bias associated with our treatment assignment strategy.

2.3 Bounding results

We also computed bounds on our estimators that relax either a linear trends or parallel trends assumptions, as in[1]. Focusing first on models that allow for linear time trends, we find that our four main results that were significant in the main analysis (mobility, USAFacts incidence, CDC incidence, and R_t) remain significant when we allow for linear time trends ("No deviation"). However, allowing for a deviation from these trends as large as any observed in the pre-period indicates that our results are sensitive to violations of this linear trend assumption.

Under a parallel trends assumption our results are, again, significant and we can rule out all but the smallest reductions in COVID-19 incidence association with campus reopenings for all outcomes except for cases resulting in hospitalization. Allowing for deviations from parallel trends that are as large as the largest deviation in the pre-period yields wider confidence intervals that typically include both positive and negative values. However, these intervals are substantially narrower than the violations from linear trends.

References

[1] Rambachan A, Roth J. An honest approach to parallel trends. Unpublished manuscript, Harvard University[99]. 2021;.
