# Supplementary material for: College openings in the United States increase mobility and COVID-19 incidence
Source: PLoS One. 2022 Aug 29;17(8):e0272820. doi: 10.1371/journal.pone.0272820 (PMC9423614; doi:10.1371/journal.pone.0272820)
Supplement: S1 Table — (PDF) [file pone.0272820.s008.pdf]

Table 1: Teaching modalities across counties

	Coarse modality		Fine modality				
Teaching modality (# counties)	In-person	Online	Fully In Person	Primarily In Person	Hybrid	Primarily Online	Fully Online
**Single-Campus counties**							
In-person (393)	1.00	0.00	0.08	0.58	0.35	0.00	0.00
Online (136)	0.00	1.00	0.00	0.00	0.00	0.88	0.12
Fully In Person (31)	1.00	0.00	1.00	0.00	0.00	0.00	0.00
Primarily In Person (226)	1.00	0.00	0.00	1.00	0.00	0.00	0.00
Hybrid (136)	1.00	0.00	0.00	0.00	1.00	0.00	0.00
Primarily Online (119)	0.00	1.00	0.00	0.00	0.00	1.00	0.00
Fully Online (17)	0.00	1.00	0.00	0.00	0.00	0.00	1.00
**Multi-Campus counties**							
In-person (159)	0.78	0.22	0.04	0.39	0.36	0.16	0.06
Online (98)	0.34	0.66	0.02	0.14	0.19	0.52	0.13
Fully In Person (6)	0.88	0.12	0.44	0.29	0.14	0.08	0.04
Primarily In Person (80)	0.82	0.18	0.03	0.60	0.19	0.14	0.04
Hybrid (73)	0.73	0.27	0.01	0.16	0.56	0.20	0.07
Primarily Online (85)	0.35	0.65	0.02	0.15	0.19	0.56	0.09
Fully Online (13)	0.28	0.72	0.01	0.08	0.19	0.30	0.42

Source–Authors' analysis of C2I data.

Notes–Values are the row fraction of campuses in each county with the teaching modality indicated by the column header. Row label indicates the teaching modality assignment of the county.
