# Supplementary material for: College openings in the United States increase mobility and COVID-19 incidence
Source: PLoS One. 2022 Aug 29;17(8):e0272820. doi: 10.1371/journal.pone.0272820 (PMC9423614; doi:10.1371/journal.pone.0272820)
Supplement: S2 Table — (PDF) [file pone.0272820.s009.pdf]

Table 2: Summary statistics on the sample.

		Primarily in-person		Primarily online	
	No college	Before	After	Before	After
Log visitors	4.17 (0.558)	4.54 (0.852)	4.95 (0.760)	4.27 (0.896)	4.64 (0.870)
Visitors	99.9 (62.8)	165.0 (171.4)	218.4 (199.7)	134.0 (147.9)	176.6 (178.5)
Daily new cases per 100,000...					
...from USAFacts	20.7 (54.8)	13.5 (49.6)	21.6 (33.1)	13.0 (19.6)	19.1 (39.3)
...from CDC	20.9 (34.5)	12.3 (14.9)	21.1 (23.4)	12.6 (14.6)	19.4 (25.3)
...resulting in hospitalization	1.156 (3.80)	0.742 (1.39)	0.949 (1.68)	0.761 (1.27)	0.847 (1.53)
...resulting in ICU admission	0.1191 (1.222)	0.0794 (0.415)	0.0905 (0.473)	0.0905 (0.308)	0.0734 (0.311)
...resulting in death	0.483 (2.548)	0.226 (0.729)	0.375 (1.067)	0.237 (0.861)	0.321 (1.057)
R_t	1.39 (1.269)	1.21 (0.637)	1.11 (0.617)	1.18 (0.840)	1.10 (0.410)
Exposure ('0000s)	—	1.68 (1.65)	1.77 (1.70)	1.33 (1.38)	1.41 (1.44)

Source—Authors' analysis of C2I data on college reopening, SafeGraph mobility data, and COVID-19 case and mortality data.
