# Supplementary material for: College openings in the United States increase mobility and COVID-19 incidence
Source: PLoS One. 2022 Aug 29;17(8):e0272820. doi: 10.1371/journal.pone.0272820 (PMC9423614; doi:10.1371/journal.pone.0272820)
Supplement: S3 Table — (PDF) [file pone.0272820.s010.pdf]

Table 3: Event study coefficients

	Log visitors	Daily new cases per 100,000 from USAFacts	Daily new cases per 100,000 from CDC	Daily new cases per 100,000 resulting in hospitalization	Daily new cases per 100,000 resulting in ICU admission	Daily new cases per 100,000 resulting in death	Rt
All							
-8	-0.108 (0.009)	-0.457 (0.666)	-0.671 (0.696)	-0.163 (0.058)	-0.005 (0.013)	-0.044 (0.026)	-0.008 (0.026)
-7	-0.095 (0.007)	-1.101 (0.579)	-0.255 (0.648)	-0.153 (0.051)	-0.008 (0.009)	-0.013 (0.026)	-0.024 (0.02)
-6	-0.066 (0.007)	-1.119 (0.551)	-0.377 (0.433)	-0.054 (0.043)	-0.004 (0.009)	-0.01 (0.023)	0.011 (0.026)
-5	-0.051 (0.006)	-1.098 (0.492)	-0.532 (0.39)	-0.042 (0.04)	0.007 (0.011)	0.028 (0.023)	-0.02 (0.018)
-4	-0.038 (0.005)	-0.693 (0.682)	-0.068 (0.313)	-0.027 (0.038)	0.022 (0.013)	0.003 (0.022)	0.008 (0.016)
-3	—	—	—	—	—	—	—
-2	0.105 (0.006)	0.107 (0.486)	0.546 (0.314)	-0.03 (0.036)	-0.006 (0.01)	0.004 (0.023)	0.002 (0.015)
-1	0.369 (0.013)	0.857 (0.533)	1.019 (0.554)	-0.022 (0.035)	0.005 (0.01)	0.006 (0.022)	0.028 (0.015)
0	0.456 (0.017)	2.628 (0.674)	2.507 (0.706)	-0.028 (0.048)	0.024 (0.011)	0.008 (0.032)	0.075 (0.018)
1	0.431 (0.017)	5.118 (1.283)	3.572 (0.872)	-0.018 (0.053)	0.013 (0.012)	0.039 (0.031)	0.081 (0.021)
2	0.408 (0.017)	6.574 (1.575)	3.921 (0.953)	0.013 (0.068)	0.003 (0.012)	0.05 (0.033)	0.068 (0.021)
3	0.413 (0.018)	5.65 (1.659)	3.099 (0.985)	0.006 (0.073)	0.014 (0.013)	0.026 (0.039)	0.005 (0.025)
4	0.405 (0.017)	6.099 (1.738)	2.945 (1.091)	-0.023 (0.075)	0.011 (0.014)	0.021 (0.04)	-0.019 (0.022)
5	0.376 (0.017)	6.178 (1.809)	2.854 (1.191)	0.011 (0.081)	-0.009 (0.013)	0.002 (0.039)	-0.012 (0.025)
6	0.341 (0.016)	6.033 (1.855)	2.64 (1.251)	-0.003 (0.089)	0 (0.014)	0.035 (0.048)	-0.034 (0.019)
7	0.327 (0.016)	6.793 (1.934)	2.662 (1.391)	0.04 (0.093)	-0.004 (0.017)	0.033 (0.049)	-0.024 (0.022)
In-Person							
-8	-0.119 (0.01)	-0.937 (0.91)	-1.195 (1.021)	-0.195 (0.083)	0.005 (0.02)	-0.087 (0.038)	-0.03 (0.034)
-7	-0.108 (0.008)	-1.479 (0.763)	-0.566 (0.942)	-0.181 (0.075)	-0.001 (0.012)	-0.042 (0.035)	-0.032 (0.025)
-6	-0.068 (0.007)	-1.459 (0.751)	-0.787 (0.621)	-0.072 (0.066)	0.009 (0.012)	-0.02 (0.03)	-0.019 (0.025)
-5	-0.053 (0.006)	-1.578 (0.65)	-0.98 (0.51)	-0.075 (0.055)	0.016 (0.014)	0.013 (0.031)	-0.031 (0.022)
-4	-0.041 (0.005)	-1.516 (0.946)	-0.276 (0.369)	-0.054 (0.044)	0.036 (0.017)	-0.023 (0.029)	-0.005 (0.017)
-3	—	—	—	—	—	—	—
-2	0.118 (0.007)	0.105 (0.677)	0.555 (0.359)	-0.002 (0.047)	0.002 (0.012)	-0.001 (0.031)	-0.003 (0.019)
-1	0.433 (0.016)	1.098 (0.665)	0.993 (0.731)	0.007 (0.049)	0.015 (0.013)	0.01 (0.028)	0.015 (0.019)
0	0.551 (0.021)	2.596 (0.723)	2.388 (0.831)	-0.045 (0.055)	0.029 (0.016)	0.003 (0.034)	0.056 (0.021)
1	0.512 (0.021)	5.972 (1.716)	3.841 (1.081)	0.041 (0.061)	0.019 (0.015)	0.06 (0.04)	0.069 (0.024)
2	0.479 (0.021)	6.901 (1.863)	3.96 (1.024)	0.051 (0.067)	0.016 (0.015)	0.052 (0.04)	0.067 (0.023)
3	0.478 (0.021)	5.813 (1.893)	2.742 (0.992)	0.064 (0.077)	0.031 (0.016)	0.024 (0.041)	0.006 (0.029)
4	0.465 (0.021)	6.119 (1.944)	2.325 (1.095)	0.025 (0.077)	0.021 (0.015)	0.04 (0.046)	-0.017 (0.024)
5	0.431 (0.02)	6.032 (2.006)	2.36 (1.192)	0.007 (0.084)	0.001 (0.015)	-0.005 (0.045)	-0.016 (0.03)
6	0.391 (0.02)	5.761 (2.024)	2.229 (1.217)	-0.014 (0.09)	0.012 (0.017)	0.005 (0.051)	-0.034 (0.022)
7	0.37 (0.02)	6.341 (2.075)	1.975 (1.366)	0.011 (0.094)	-0.001 (0.018)	0.002 (0.052)	-0.03 (0.023)
Online							
-8	-0.097 (0.017)	0.458 (0.838)	-0.516 (0.811)	-0.156 (0.09)	-0.028 (0.017)	0.002 (0.027)	0.031 (0.035)
-7	-0.079 (0.014)	-0.668 (0.918)	-0.384 (0.751)	-0.148 (0.078)	-0.024 (0.016)	0.017 (0.044)	-0.015 (0.033)
-6	-0.068 (0.014)	-0.957 (0.764)	-0.32 (0.629)	-0.052 (0.058)	-0.03 (0.015)	-0.014 (0.04)	0.076 (0.061)
-5	-0.052 (0.013)	-0.467 (0.705)	-0.161 (0.667)	0.002 (0.064)	-0.008 (0.016)	0.034 (0.033)	0.013 (0.026)
-4	-0.036 (0.008)	1.02 (0.653)	0.111 (0.596)	0.019 (0.07)	-0.003 (0.015)	0.045 (0.03)	0.051 (0.029)
-3	—	—	—	—	—	—	—
-2	0.092 (0.009)	0.374 (0.515)	0.576 (0.639)	-0.074 (0.055)	-0.026 (0.014)	0.02 (0.025)	0.015 (0.022)
-1	0.28 (0.019)	0.86 (0.971)	1.33 (0.922)	-0.058 (0.06)	-0.019 (0.014)	0.007 (0.038)	0.058 (0.024)
0	0.322 (0.022)	3.386 (1.28)	3.085 (1.393)	0.025 (0.101)	0.008 (0.012)	0.03 (0.071)	0.107 (0.028)
1	0.314 (0.021)	4.583 (1.621)	3.335 (1.484)	-0.087 (0.091)	-0.009 (0.015)	-0.002 (0.051)	0.11 (0.033)
2	0.308 (0.023)	7.147 (1.995)	3.912 (1.827)	-0.035 (0.119)	-0.027 (0.017)	0.041 (0.055)	0.076 (0.033)
3	0.321 (0.023)	6.509 (2.105)	3.805 (1.876)	-0.088 (0.113)	-0.026 (0.016)	0.029 (0.079)	0.013 (0.034)
4	0.321 (0.025)	7.409 (2.219)	4.093 (2.075)	-0.094 (0.119)	-0.015 (0.024)	-0.026 (0.07)	-0.02 (0.032)
5	0.299 (0.024)	7.862 (2.328)	3.731 (2.107)	0.025 (0.125)	-0.032 (0.018)	0.001 (0.062)	0.002 (0.03)
6	0.268 (0.023)	7.874 (2.352)	3.343 (2.187)	0.013 (0.136)	-0.027 (0.02)	0.062 (0.085)	-0.026 (0.027)
7	0.266 (0.024)	9.132 (2.574)	4.081 (2.362)	0.106 (0.148)	-0.014 (0.026)	0.074 (0.082)	0 (0.033)

Source–Authors' analysis of C2I data, SafeGraph mobility data, and CDC COVID-19 case data.
Notes–Estimates are aggregated treatment effects from generalized difference-in-differences regressions for each week, relative to reopening. Standard error, clustered on county, reported in round brackets. Each column in each panel is from a separate specification.
