# Supplementary material for: College openings in the United States increase mobility and COVID-19 incidence
Source: PLoS One. 2022 Aug 29;17(8):e0272820. doi: 10.1371/journal.pone.0272820 (PMC9423614; doi:10.1371/journal.pone.0272820)
Supplement: S4 Table — (PDF) [file pone.0272820.s011.pdf]

Table 4: Difference-in-difference regressions demonstrating the effect of reopening college campuses on mobility and COVID-19 incidence and sequelae.

	Log visitors	Daily new cases per 100,000 from USAFacts	Daily new cases per 100,000 from CDC	Daily new hospitalized cases per 100,000	Daily new cases in ICU per 100,000	Daily new deaths per 100,000	Rt
Baseline	0.322 (0.015)	4.885 (1.004)	2.729 (0.772)	0.023 (0.052)	0.020 (0.010)	0.027 (0.031)	0.056 (0.016)
	[0.293, 0.351]	[2.918, 6.853]	[1.215, 4.242]	[-0.079, 0.125]	[-0.001, 0.040]	[-0.034, 0.088]	[0.025, 0.087]
	{p<0.001}	{p<0.001}	{p<0.001}	{p=0.661}	{p=0.057}	{p=0.391}	{p<0.001}
In-person	0.388 (0.019)	5.215 (1.129)	2.677 (0.826)	0.029 (0.055)	0.022 (0.014)	0.036 (0.029)	0.053 (0.018)
	[0.351, 0.424]	[3.002, 7.428]	[1.059, 4.295]	[-0.079, 0.137]	[-0.005, 0.048]	[-0.020, 0.092]	[0.018, 0.087]
	{p<0.001}	{p<0.001}	{p=0.001}	{p=0.593}	{p=0.106}	{p=0.208}	{p=0.003}
Online	0.225 (0.017)	5.032 (1.417)	2.958 (1.535)	0.027 (0.084)	0.013 (0.011)	0.005 (0.069)	0.062 (0.027)
	[0.191, 0.259]	[2.254, 7.810]	[-0.050, 5.967]	[-0.137, 0.192]	[-0.010, 0.035]	[-0.130, 0.139]	[0.009, 0.115]
	{p<0.001}	{p<0.001}	{p=0.054}	{p=0.745}	{p=0.271}	{p=0.944}	{p=0.022}
In-person = Online, $\chi^2(2)$ (p-value)	166.04 (<0.001)	0.05 (0.830)	0.03 (0.867)	0 (0.979)	0.33 (0.568)	0.19 (0.667)	0.23 (0.635)
Week(s) relative to reopening							
Week prior	0.276 (0.014)	1.720 (0.790)	0.714 (0.758)	-0.023 (0.065)	0.016 (0.017)	-0.013 (0.037)	0.045 (0.016)
	[0.249, 0.303]	[0.172, 3.268]	[-0.772, 2.200]	[-0.151, 0.104]	[-0.017, 0.049]	[-0.085, 0.059]	[0.013, 0.077]
	{p<0.001}	{p=0.029}	{p=0.346}	{p=0.718}	{p=0.335}	{p=0.723}	{p=0.006}
Weeks 0-1	0.350 (0.018)	4.736 (0.903)	2.734 (0.925)	-0.025 (0.073)	0.030 (0.022)	0.005 (0.040)	0.095 (0.017)
	[0.315, 0.386]	[2.966, 6.506]	[0.921, 4.548]	[-0.168, 0.119]	[-0.013, 0.073]	[-0.074, 0.084]	[0.062, 0.128]
	{p<0.001}	{p<0.001}	{p=0.003}	{p=0.734}	{p=0.178}	{p=0.906}	{p<0.001}
Weeks 2-3	0.318 (0.019)	6.975 (1.513)	3.205 (1.093)	0.008 (0.090)	0.020 (0.023)	0.019 (0.044)	0.054 (0.020)
	[0.281, 0.354]	[4.009, 9.940]	[1.062, 5.348]	[-0.168, 0.183]	[-0.025, 0.064]	[-0.068, 0.106]	[0.015, 0.093]
	{p<0.001}	{p<0.001}	{p=0.003}	{p=0.932}	{p=0.386}	{p=0.666}	{p=0.007}
Weeks 4+	0.269 (0.017)	7.139 (1.744)	2.470 (1.298)	0.005 (0.100)	0.011 (0.023)	0.004 (0.052)	-0.005 (0.017)
	[0.235, 0.303]	[3.721, 10.557]	[-0.074, 5.014]	[-0.190, 0.200]	[-0.035, 0.056]	[-0.097, 0.105]	[-0.039, 0.028]
	{p<0.001}	{p<0.001}	{p=0.057}	{p=0.960}	{p=0.646}	{p=0.941}	{p=0.755}
Equality across weeks 0-4+, $\chi^2(2)$ (p-value)	165.99 (<0.001)	23.05 (<0.001)	10.41 (0.015)	0.63 (0.889)	4.36 (0.225)	1.53 (0.677)	42.95 (<0.001)
Terciles of COVID-19 exposure							
1st tercile	0.280 (0.018)	3.887 (0.952)	2.215 (0.694)	0.092 (0.059)	0.016 (0.013)	0.064 (0.028)	0.085 (0.039)
	[0.244, 0.316]	[2.021, 5.753]	[0.854, 3.575]	[-0.023, 0.207]	[-0.010, 0.042]	[0.009, 0.119]	[0.009, 0.162]
	{p<0.001}	{p<0.001}	{p=0.001}	{p=0.117}	{p=0.226}	{p=0.023}	{p=0.028}
2nd tercile	0.272 (0.018)	5.206 (1.266)	2.108 (1.162)	0.044 (0.082)	0.018 (0.016)	-0.012 (0.064)	0.019 (0.020)
	[0.236, 0.308]	[2.726, 7.687]	[-0.169, 4.385]	[-0.117, 0.204]	[-0.013, 0.049]	[-0.136, 0.113]	[-0.020, 0.058]
	{p<0.001}	{p<0.001}	{p=0.070}	{p=0.592}	{p=0.263}	{p=0.854}	{p=0.351}
3rd tercile	0.381 (0.026)	5.953 (1.487)	4.303 (1.576)	-0.026 (0.072)	0.019 (0.019)	0.045 (0.037)	0.068 (0.020)
	[0.330, 0.431]	[3.039, 8.867]	[1.215, 7.392]	[-0.168, 0.116]	[-0.018, 0.055]	[-0.027, 0.118]	[0.028, 0.107]
	{p<0.001}	{p<0.001}	{p=0.006}	{p=0.724}	{p=0.315}	{p=0.218}	{p<0.001}
Equality across terciles, $\chi^2(2)$ (p-value)	52.71 (<0.001)	8.9 (0.012)	1.72 (0.424)	2.81 (0.246)	0.02 (0.991)	1.3 (0.521)	10.95 (0.004)
Terciles of college student share							
1st tercile	0.193 (0.014)	2.085 (1.480)	-0.077 (0.902)	-0.089 (0.087)	0.009 (0.017)	0.001 (0.040)	0.023 (0.025)
	[0.165, 0.222]	[-0.815, 4.986]	[-1.846, 1.691]	[-0.260, 0.082]	[-0.024, 0.042]	[-0.076, 0.078]	[-0.026, 0.072]
	{p<0.001}	{p=0.159}	{p=0.932}	{p=0.306}	{p=0.608}	{p=0.980}	{p=0.351}
2nd tercile	0.287 (0.020)	2.060 (1.533)	-0.220 (1.077)	0.035 (0.081)	0.021 (0.012)	0.004 (0.072)	0.023 (0.024)
	[0.247, 0.327]	[-0.944, 5.064]	[-2.331, 1.892]	[-0.124, 0.194]	[-0.002, 0.044]	[-0.136, 0.145]	[-0.024, 0.070]
	{p<0.001}	{p=0.179}	{p=0.839}	{p=0.664}	{p=0.075}	{p=0.953}	{p=0.331}
3rd tercile	0.463 (0.026)	11.434 (1.214)	9.018 (1.598)	0.148 (0.070)	0.020 (0.021)	0.077 (0.039)	0.126 (0.026)
	[0.411, 0.515]	[9.054, 13.813]	[5.885, 12.151]	[0.010, 0.287]	[-0.021, 0.061]	[0.001, 0.154]	[0.076, 0.177]
	{p<0.001}	{p<0.001}	{p<0.001}	{p=0.035}	{p=0.335}	{p=0.047}	{p<0.001}
Equality across terciles, $\chi^2(2)$ (p-value)	243.4 (<0.001)	98.83 (<0.001)	27.48 (<0.001)	5.31 (0.070)	0.43 (0.805)	1.95 (0.376)	28.72 (<0.001)

Source—Authors' analysis of C2I data on college reopening, SafeGraph mobility data, and COVID-19 case and mortality data.
Notes—Estimates are aggregated treatment effects from generalized difference-in-differences regressions for the first 28 days following reopening. "Primarily in-person" includes "Fully in-person" and "Hybrid"; "Primarily online" includes "Fully online". Exposure is the 7-day COVID-19 incidence in source counties for each college two weeks prior to reopening, weighted by the change in movement to the campus from each county. Column titles indicate the dependent variable; each panel is a separate specification. Z-statistics in parentheses following point estimates, 95% confidence intervals in square brackets, and p-values in curly brackets.
