# Supplementary material for: College openings in the United States increase mobility and COVID-19 incidence
Source: PLoS One. 2022 Aug 29;17(8):e0272820. doi: 10.1371/journal.pone.0272820 (PMC9423614; doi:10.1371/journal.pone.0272820)
Supplement: S5 Table — (PDF) [file pone.0272820.s012.pdf]

Table 5: Age-specific difference-in-differences demonstrates that the increase in incidence of COVID-19 was isolated to people between 10 and 29 years of age, which encompasses college-age individuals

	Daily new cases per 100,000	Daily new hospitalized cases per 100,000	Daily new cases in ICU per 100,000	Daily new deaths per 100,000
0 - 9	-0.293 (0.361)	0.008 (0.017)	0.007 (0.006)	-0.003 (0.002)
	[-1.000, 0.414]	[-0.026, 0.042]	[-0.006, 0.019]	[-0.006, 0.000]
	{p=0.417}	{p=0.631}	{p=0.309}	{p=0.091}
10 - 19	8.027 (1.278)	0.084 (0.034)	0.004 (0.003)	-0.001 (0.002)
	[5.522, 10.531]	[0.018, 0.149]	[-0.001, 0.010]	[-0.004, 0.003]
	{p<0.001}	{p=0.013}	{p=0.132}	{p=0.644}
20 - 29	7.378 (1.395)	0.062 (0.053)	-0.003 (0.012)	0.005 (0.006)
	[4.643, 10.113]	[-0.042, 0.167]	[-0.026, 0.021]	[-0.006, 0.016]
	{p<0.001}	{p=0.241}	{p=0.816}	{p=0.337}
30 - 49	-0.129 (0.909)	0.029 (0.064)	0.012 (0.010)	0.021 (0.011)
	[-1.912, 1.653]	[-0.096, 0.154]	[-0.009, 0.032]	[-0.001, 0.042]
	{p=0.887}	{p=0.645}	{p=0.254}	{p=0.059}
50 - 69	0.283 (0.650)	0.036 (0.094)	0.044 (0.023)	0.026 (0.039)
	[-0.991, 1.558]	[-0.149, 0.220]	[-0.001, 0.088]	[-0.050, 0.103]
	{p=0.663}	{p=0.704}	{p=0.054}	{p=0.500}
70+	0.746 (0.893)	0.024 (0.242)	0.063 (0.063)	0.185 (0.209)
	[-1.003, 2.496]	[-0.450, 0.497]	[-0.060, 0.186]	[-0.224, 0.594]
	{p=0.403}	{p=0.922}	{p=0.316}	{p=0.375}
$\chi^2(5)$	67.21 (<0.001)	5.28 (0.382)	5.15 (0.398)	7.57 (0.182)

Source—Authors' analysis of C2I data, SafeGraph mobility data, and CDC COVID-19 case data.
Notes—Estimates are aggregated treatment effects from generalized difference-in-differences regressions. Column titles indicate the class of the dependent variable and the row label indicates the age group. Standard errors in parentheses following point estimates, 95% confidence intervals in square brackets, and p-values in curly brackets. χ^2 tests for equality across age groups are reported in the bottom row.
