# Supplementary material for: College openings in the United States increase mobility and COVID-19 incidence
Source: PLoS One. 2022 Aug 29;17(8):e0272820. doi: 10.1371/journal.pone.0272820 (PMC9423614; doi:10.1371/journal.pone.0272820)
Supplement: S6 Table — (PDF) [file pone.0272820.s013.pdf]

Table 6: Age-specific means of the dependent variables

	Incidence		Hospitalizations		ICU admissions		Deaths	
	Pre-period	Post-period	Pre-period	Post-period	Pre-period	Post-period	Pre-period	Post-period
USAFacts	13.32	21.54						
	(40.60)	(36.10)						
CDC (All ages)	12.39	21.29	0.74	0.94	0.08	0.09	0.23	0.37
	(14.99)	(24.51)	(1.33)	(1.66)	(0.38)	(0.43)	(0.77)	(1.09)
0 - 9	4.35	6.48	0.06	0.06	0.00	0.00	0.00	0.00
	(11.36)	(12.95)	(0.81)	(0.76)	(0.16)	(0.26)	(0.03)	(0.02)
10 - 19	10.28	22.22	0.08	0.11	0.01	0.00	0.00	0.00
	(16.75)	(34.32)	(0.79)	(1.02)	(0.31)	(0.15)	(0.04)	(0.13)
20 - 29	19.19	30.22	0.28	0.25	0.01	0.01	0.01	0.01
	(26.81)	(40.36)	(1.46)	(1.53)	(0.26)	(0.52)	(0.18)	(0.28)
30 - 49	15.44	23.49	0.55	0.54	0.05	0.04	0.04	0.04
	(23.79)	(33.09)	(1.97)	(2.27)	(0.56)	(0.59)	(0.72)	(0.55)
50 - 69	11.22	19.49	1.07	1.29	0.14	0.14	0.24	0.29
	(15.63)	(25.58)	(2.80)	(3.33)	(0.92)	(0.99)	(1.46)	(1.49)
70+	10.40	20.42	2.63	4.03	0.30	0.38	1.49	2.63
	(20.34)	(35.03)	(6.89)	(9.70)	(2.02)	(2.65)	(5.27)	(8.57)

Source–Authors' analysis of CDC and USAFacts data.
Notes–Values are the mean and standard deviation (parentheses) for the indicated variable, measured per 100,000 people. Sample restricted to counties with a college.
