# Supplementary material for: College openings in the United States increase mobility and COVID-19 incidence
Source: PLoS One. 2022 Aug 29;17(8):e0272820. doi: 10.1371/journal.pone.0272820 (PMC9423614; doi:10.1371/journal.pone.0272820)
Supplement: S7 Table — (PDF) [file pone.0272820.s014.pdf]

Table 7: Robustness checks discussed in section 1

	Log visitors	Daily new cases per 100,000 from USAFacts	Daily new cases per 100,000 from CDC	Daily new cases per 100,000 resulting in hospitalization	Daily new cases per 100,000 resulting in ICU admission	Daily new cases per 100,000 resulting in death	Rt
Baseline	0.322 (0.015)	4.89 (1.00)	2.73 (0.77)	0.023 (0.052)	0.020 (0.010)	0.027 (0.031)	0.056 (0.016)
	[0.293, 0.351]	[2.92, 6.85]	[1.22, 4.24]	[-0.079,0.125]	[-0.001,0.040]	[-0.034,0.088]	[0.025, 0.087]
	{p<0.001}	{p<0.001}	{p<0.001}	{p=0.661}	{p=0.057}	{p=0.391}	{p<0.001}
Colleges only	0.269 (0.060)	2.51 (1.83)	-3.38 (1.93)	0.152 (0.092)	0.098 (0.038)	0.016 (0.080)	0.051 (0.039)
	[0.152, 0.386]	[-1.09, 6.10]	[-7.16, 0.40]	[-0.029,0.332]	[0.024, 0.172]	[-0.140,0.172]	[-0.025,0.127]
	{p<0.001}	{p=0.171}	{p=0.079}	{p=0.099}	{p=0.009}	{p=0.840}	{p=0.187}
Single campus		5.30 (0.85)	3.17 (0.97)	0.025 (0.056)	0.017 (0.013)	0.013 (0.043)	0.069 (0.019)
		[3.62, 6.97]	[1.28, 5.07]	[-0.09, 0.14]	[-0.009,0.043]	[-0.072,0.098]	[0.032, 0.106]
		{p<0.001}	{p=0.001}	{p=0.656}	{p=0.197}	{p=0.769}	{p<0.001}
Smoothness restrictions (linear trends)							
No deviation	[0.355, 0.419]	[4.07, 7.93]	[1.80, 4.92]	[-0.073,0.133]	[-0.006,0.028]	[-0.005,0.094]	[0.032, 0.085]
Deviation	[0.099, 0.755]	[-10.33,20.31]	[-8.82,15.37]	[-1.351,1.337]	[-0.524,0.551]	[-0.789, 0.85]	[-0.938,1.052]
Relative magnitude restriction (parallel trends)							
No deviation	[0.394, 0.459]	[2.70, 7.26]	[1.73, 4.78]	[-0.110,0.100]	[-0.006,0.033]	[-0.025,0.088]	[0.025, 0.09]
Deviation	[0.212, 0.647]	[-4.50, 12.54]	[-1.41, 8.24]	[-0.772,0.723]	[-0.177,0.213]	[-0.329,0.377]	[-0.294,0.420]

Source–Authors' analysis of C2I data, SafeGraph mobility data, and CDC COVID-19 case data.

Notes–Estimates are aggregated treatment effects from generalized difference-in-differences regressions for the first 8 weeks following reopening. Column titles indicate the dependent variable; each panel is a separate specification. "Colleges only" restricts the sample to Census Block Groups and counties with a college campus. "Single campus" restricts to counties without a college campus or with only one collect campus. Standard errors in parentheses following point estimates, 95% confidence intervals in square brackets, and p-values in curly brackets. "Smoothness restrictions" bound the effect of deviations from linear trends using the approach in Rambachan and Roth[1]; "No deviation" assumes that linear trends in the pre-period continue into the post-period, while "Deviation" allows for deviations from a linear trend as large as the largest deviation observed in the pre-period. "Relative magnitude restrictions" bound the effect of deviations from a parallel trends assumption, with "no deviation" assuming that parallel trends holds in the post-period, regardless of any differential trends in the pre-period, while "Deviation" allows for a deviation from parallel trends that is as large as the largest deviation from parallel trends observed in the pre-period.
