# Supplementary material for: College openings in the United States increase mobility and COVID-19 incidence
Source: PLoS One. 2022 Aug 29;17(8):e0272820. doi: 10.1371/journal.pone.0272820 (PMC9423614; doi:10.1371/journal.pone.0272820)
Supplement: S8 Table — (PDF) [file pone.0272820.s015.pdf]

Table 8: Weekly and daily pre-trend tests

	Log visitors	Daily new cases per 100,000 from USAFacts	Daily new cases per 100,000 from CDC	Daily new cases per 100,000 resulting in hospitalization	Daily new cases per 100,000 resulting in ICU admission	Daily new cases per 100,000 resulting in death	Rt
**Weekly pre-test**							
All	174.69 (<0.001)	5.93 (0.313)	3.41 (0.637)	10.54 (0.061)	7.35 (0.196)	3.85 (0.571)	6.24 (0.284)
In-person	198.88 (<0.001)	6.46 (0.264)	4.93 (0.424)	7.42 (0.191)	7.71 (0.173)	4.91 (0.427)	3.17 (0.674)
Online	35.1 (<0.001)	5.74 (0.333)	0.58 (0.989)	4.83 (0.436)	4.36 (0.498)	2.9 (0.716)	8.06 (0.153)
Fully in-person	10.52 (0.062)	3.17 (0.674)	1.35 (0.930)	5.11 (0.403)	9 (0.109)	7.12 (0.212)	8.93 (0.112)
Primarily in-person	105.41 (<0.001)	4.05 (0.542)	4.68 (0.456)	11.87 (0.037)	5.49 (0.359)	3.39 (0.639)	1.69 (0.890)
Hybrid	95.59 (<0.001)	3.37 (0.643)	3.57 (0.613)	2.11 (0.834)	9.81 (0.081)	6.07 (0.299)	2.56 (0.767)
Primarily online	56.73 (<0.001)	4.68 (0.457)	1.31 (0.934)	4 (0.550)	5.74 (0.332)	4.5 (0.480)	6.35 (0.274)
Fully online	1.99 (0.851)	5.72 (0.334)	1.35 (0.930)	3.47 (0.628)	3.3 (0.654)	8.28 (0.142)	10.91 (0.053)
Single Campus		0.99 (0.963)	3.88 (0.566)	6.64 (0.249)	5.37 (0.372)	5 (0.416)	2.14 (0.829)
Colleges only	59.97 (<0.001)	13.75 (0.017)	3.64 (0.603)	7.42 (0.191)	3.05 (0.692)	3.92 (0.560)	5.04 (0.411)
**Daily pre-test**							
All	857.49 (<0.001)	36.93 (0.380)	48.23 (0.068)	49.11 (0.057)	49.46 (0.053)	26.08 (0.863)	31.83 (0.622)
In-person	788.68 (<0.001)	43.32 (0.158)	44.73 (0.126)	36.38 (0.404)	48.26 (0.067)	38.27 (0.323)	39.85 (0.263)
Online	409 (<0.001)	57.03 (0.011)	55.04 (0.017)	56.98 (0.011)	53.06 (0.026)	40.28 (0.248)	50.52 (0.043)
Fully in-person	765.29 (<0.001)	137.52 (<0.001)	787.56 (<0.001)	456.16 (<0.001)	162.42 (<0.001)	228.87 (<0.001)	128.74 (<0.001)
Primarily in-person	449.1 (<0.001)	39.11 (0.290)	81.98 (<0.001)	56.44 (0.012)	48.2 (0.068)	52.14 (0.031)	29.98 (0.709)
Hybrid	625.09 (<0.001)	44.73 (0.126)	58.32 (0.008)	57.16 (0.010)	41.27 (0.215)	65.75 (0.001)	45.49 (0.110)
Primarily online	458.12 (<0.001)	55.5 (0.015)	72.92 (<0.001)	91.06 (<0.001)	62.6 (0.003)	46.46 (0.093)	46.61 (0.091)
Fully online	228.81 (<0.001)	116.29 (<0.001)	367 (<0.001)	187.22 (<0.001)	46.27 (0.096)	227.16 (<0.001)	263.42 (<0.001)
Single Campus		33.53 (0.539)	55.87 (0.014)	45.17 (0.116)	62.67 (0.003)	25.48 (0.881)	24.26 (0.914)
Colleges only	118.49 (<0.001)	55.11 (0.017)	57.69 (0.009)	48.64 (0.062)	48.95 (0.059)	26.75 (0.840)	37.78 (0.343)

Source–Authors' analysis of C2I data, SafeGraph mobility data, and CDC COVID-19 case data.
Notes–Values are χ^2 test statistics for the existence of pre-trends over the period from 7 to 3 weeks before reopening ("weekly" tests) or 56 to 15 days ("daily" tests). P-values reported in curly brackets. Test statistics are robust to clustering on county.
