# Supplementary material for: College openings in the United States increase mobility and COVID-19 incidence
Source: PLoS One. 2022 Aug 29;17(8):e0272820. doi: 10.1371/journal.pone.0272820 (PMC9423614; doi:10.1371/journal.pone.0272820)
Supplement: Attachment — Submitted filename: Rebuttal.pdf [file pone.0272820.s016.pdf]

1. Please ensure that your manuscript meets PLOS ONE's style requirements, including those for file naming. The PLOS ONE style templates can be found at https://journals.plos.org/plosone/s/file?id=wjVg/PLOSOne_formatting_sample_main_body.pdf and https://journals.plos.org/plosone/s/file?id=ba62/PLOSOne_formatting_sample_title_authors_affiliations.pdf

We have revised the naming pattern for the supplementary files to comply with journal requirements, reordered materials and made other edits to comply with the templates.

2. Please provide additional details regarding participant consent. In the ethics statement in the Methods and online submission information, please ensure that you have specified (1) whether consent was informed and (2) what type you obtained (for instance, written or verbal, and if verbal, how it was documented and witnessed). If your study included minors, state whether you obtained consent from parents or guardians. If the need for consent was waived by the ethics committee, please include this information.

If you are reporting a retrospective study of medical records or archived samples, please ensure that you have discussed whether all data were fully anonymized before you accessed them and/or whether the IRB or ethics committee waived the requirement for informed consent. If patients provided informed written consent to have data from their medical records used in research, please include this information

We have expanded our note on IRB approval to read "The University of North Carolina at Greensboro Institutional Review Board reviewed and approved our use of the CDC data and waived the requirement for informed consent on the part of individuals in the dataset."

Once you have amended this/these statement(s) in the Methods section of the manuscript, please add the same text to the "Ethics Statement" field of the submission form (via "Edit Submission").

For additional information about PLOS ONE ethical requirements for human subjects research, please refer to http://journals.plos.org/plosone/s/submission-guidelines#loc-human-subjects-research.

3. In your Data Availability statement, you have not specified where the minimal data set underlying the results described in your manuscript can be found. PLOS defines a study's minimal data set as the underlying data used to reach the conclusions drawn in the manuscript and any additional data required to replicate the reported study findings in their entirety. All PLOS journals require that the minimal data set be made fully available. For more information about our data policy, please see http://journals.plos.org/plosone/s/data-availability.

"Upon re-submitting your revised manuscript, please upload your study's minimal underlying data set as either Supporting Information files or to a stable, public repository and include the relevant URLs, DOIs, or accession numbers within your revised cover letter. For a list of acceptable repositories, please see http://journals.plos.org/plosone/s/data-availability#loc-recommended-repositories. Any potentially identifying patient information must be fully anonymized.

Important: If there are ethical or legal restrictions to sharing your data publicly, please explain these restrictions in detail. Please see our guidelines for more information on what we consider unacceptable restrictions to publicly sharing data: http://journals.plos.org/plosone/s/data-availability#loc-unacceptable-data-access-restrictions. Note that it is not acceptable for the authors to be the sole named individuals responsible for ensuring data access.

We will update your Data Availability statement to reflect the information you provide in your cover letter.

4. We note that Supplementary Figures 1 to 3 in your submission contain [map/satellite] images which may be copyrighted. All PLOS content is published under the Creative Commons Attribution License (CC BY 4.0), which means that the manuscript, images, and Supporting Information files will be freely available online, and any third party is permitted to access, download, copy, distribute, and use these materials in any way, even commercially, with proper attribution. For these reasons, we cannot publish previously copyrighted maps or satellite images created using proprietary data, such as Google software (Google Maps, Street View, and Earth). For more information, see our copyright guidelines: http://journals.plos.org/plosone/s/licenses-and-copyright.

We require you to either (1) present written permission from the copyright holder to publish these figures specifically under the CC BY 4.0 license, or (2) remove the figures from your submission:

All maps were produced using Census Bureau TIGRIS shapefiles, which are in the public domain. We have included a source note crediting the TIGRIS program for the shapefile.

Reviewer #1:

1. **The authors have an innovative paper on college reopening in Fall 2020. They show significant effects on mobility -- almost by definition mechanical effects since college campus are nearly vacant in summer -- using SafeGraph data. They also show significant increases in cases -- somewhat invariant to teaching modality -- at the county level relative to non-college counties. The paper does important diagnostics with generalized difference-in-differences / event studies. The paper is well written.**

   There are several important avenues to explore in a revision.

   Response: We appreciate the reviewer's view on the paper.

2. **First, and most importantly, the most noteworthy and credible results are that college reopenings increased COVID-19 cases in the community (county). Yet the parameterization of "reopening" is challenging for two key reasons.**

a. **One reason is that colleges vary tremendously in size (some large public universities have 50,000+ students while small liberal arts colleges might have 1,000 or so students), so the dosage within a community varies tremendously. Related, colleges size within a community matters a lot too -- colleges are a dominant shift in population in many "college towns" but may be insignificant in large urban centers.**
   Response: We agree that the “dose” of college reopening is likely to vary across counties. We have added an analysis to address this issue that allows for heterogeneous effects by terciles of the share of total college enrollment in the county to population. These analyses demonstrate the expected “dose-response” relationship, with the third tercile demonstrating significant differences from the other two terciles. We discuss these analyses in the main text (“Difference-in-differences”) section and discuss the single campus sample as a robustness check.

b. **The second reason is that the authors focus on the first college reopening within the community. This is likely uncontroversial for small rural communities where there is one college, but is certainly problematic for analyzing COVID-19 spread within large urban centers (which can have dozens of colleges). There are some clear suggestions that can address these specific concerns. We'd expect the models of COVID cases, hospitalizations, etc., to have stronger effects in communities where student populations are an important part of county populations (e.g., I'm guess more rural settings). In addition, it appears that in "college counties" there are on average 2 colleges per county. But that almost certainly masks many rural counties with just 1 college, and some highly urban counties with 30+ colleges. By focusing on counties with a small number of colleges, the "first opening" measure would be more convincing.**
   Response: We again agree with the reviewer and have included analyses omitting counties with more than one campus.

3. **Second, there was little mention of K-12 reopenings for Fall 2020. This must be addressed empirically as well; some states (e.g., Florida, Texas, etc.) were very aggressive about reopening public schools, while other states stayed online in Fall 2020. In principle, college reopenings in states (or counties) where K-12 remained online would provide the most convincing evidence on colleges; otherwise, there are natural concerns about similar timing of these two potential spreaders of COVID. It might be the case that the "no college" counties serve as a control for public school reopenings, but the case would need to be convincingly made.**
   Response: We have added a control for mobility to K-12 schools, which provides a dynamic alternative to an indicator for schools being open, or not, and accommodates differences in teaching modalities across schools.

4. **Third, in the introduction the authors say "As we approach Fall 2021". They should update. The conclusion seems to be updated to 2022.**
   Response: Thank you, we have updated that language.

5. **Overall, this is a terrific paper on a controversial topic that generates extreme reactions. The authors' findings make a lot of sense (although some of the extreme outcomes like mortality seem to increase relatively quickly compared to the lag we might expect from cases -- especially if spread starts among the young). However, the exploration I suggest isn't just "robustness" or footnote suggestions (in my view). Trying to characterize college reopenings in large urban areas is really hard given the multiplicity of colleges and differences in start dates. And even if colleges are areas of extreme transmission, some colleges are really small.**
   Response: We appreciate these kind remarks. We have explicitly discussed two approaches to handling variation in “exposure” to college reopenings in the paper: i) terciles of the ratio of (total) enrollment to population; and ii) restricting to counties with a single campus. We prefer the former metric since it captures dose more precisely, while the latter resolves measurement error in the reopening type categorization.

Review #2:

6. **This study contributes to an important debate on the role of college reopening policies in mitigating the transmission of COVID-19 infections. The authors use data from a variety of sources to evaluate the association between college re-openings and mobility and COVID-19 related outcomes. The study is well done. I have some minor suggestions:**

   Response: Thank you. We appreciate the reviewer’s careful and thoughtful review of our paper.

7. **In the introduction (paragraph 3), the authors write “As we approach Fall 2021, with expected mass movement events in the US …” Given that we are now in Spring 2022, this discussion should be updated to reflect the current situation. Many of these arguments remain relevant today.**

   Response: Thank you! We have updated the introduction and discussion to reflect the environment as of Spring 22.

8. **Is the natural log of county population the only variable that is included in the difference-in-differences model? Clarify whether other covariates are included in the propensity score.**

   Response: In the original manuscript that is correct. In the revision, Reviewer #1

requested that we also control for K-12 school status, so we have also included as a control mobility to K-12 schools in the county.

9. **The discussion and/or conclusion sections should acknowledge the presence of pre-trends in mobility, which limits the causal interpretation of the estimates. Clarify that a causal interpretation is only valid under the assumption that any pre-trends are fully captured by linear trends.**

   Response: We mention this limitation in the discussion section and include a discussion of results accounting for violations of the parallel trends assumption in the SI..

10. **Given that the sample includes only 70% of four-year colleges, are counties with colleges not included in the C2i data dropped from the sample? In other words, does the control group include counties with colleges for which you do not have opening dates? This should be clarified.**

    Response: As a baseline we have included all counties in our sample, so the control group includes colleges for which we do not have reopening dates. Our sample includes all degree granting, 4-year colleges that receive federal funding and enroll first-time, full-time undergraduates. We have added the sentence “Counties with colleges that were not in our sample were included in the control group” to the methods section to clarify that these counties are treated as controls.
